# Internet of Everything (IoE) Taxonomies: A Survey and a Novel Knowledge-Based Taxonomy

**DOI:** 10.3390/s21020568

**Published:** 2021-01-14

**Authors:** Viviane Cunha Farias da Costa, Luiz Oliveira, Jano de Souza

**Affiliations:** 1 Systems Engineering and Computer Science Program/COPPE, Federal University of Rio de Janeiro, Rio de Janeiro 21941-590, Brazil; lfoliveira@cos.ufrj.br (L.O.); jano@cos.ufrj.br (J.d.S.); 2 Federal Institute of Rio de Janeiro, Niterói Campus, Niterói 24315-375, Brazil

**Keywords:** Internet of everything, Internet of things, IoE, IoT, taxonomy, sensors, big-data, knowledge

## Abstract

The paradigm of the Internet of everything (IoE) is advancing toward enriching people’s lives by adding value to the Internet of things (IoT), with connections among people, processes, data, and things. This paper provides a survey of the literature on IoE research, highlighting concerns in terms of intelligence services and knowledge creation. The significant contributions of this study are as follows: (1) a systematic literature review of IoE taxonomies (including IoT); (2) development of a taxonomy to guide the identification of critical knowledge in IoE applications, an in-depth classification of IoE enablers (sensors and actuators); (3) validation of the defined taxonomy with 50 IoE applications; and (4) identification of issues and challenges in existing IoE applications (using the defined taxonomy) with regard to insights about knowledge processes. To the best of our knowledge, and taking into consideration the 76 other taxonomies compared, this present work represents the most comprehensive taxonomy that provides the orchestration of intelligence in network connections concerning knowledge processes, type of IoE enablers, observation characteristics, and technological capabilities in IoE applications.

## 1. Introduction

The Internet of everything (IoE) is a term that was first defined by CISCO in 2012 [1] as a network of networks that reunites people, processes, data, and things in network connections more significant and valuable than ever [2,3,4]. While the Internet of things (IoT) is a dynamic global network infrastructure [5] concerned about things (i.e., physical devices, accessed through the Internet), IoE lays an upper foundation over IoT and is concerned with intelligent network connections and technologies [6,7,8,9].

IoE supports creating new capacities, better-off competencies, and outstanding economic opportunities for businesses and society [1]. For Fiaidhi and Mohammed [10], IoE expands on the IoT concept by connecting devices and people in one network. Beyond the concept of IoT, the IoE paradigm covers a wide range of Internet-based concepts; for example, the Internet of people (IoP), which considers social networks and connections among people; and the industrial Internet (II), which is focused on data of interest to industry [7]. The concept of IoE incorporates nanosensors in diverse objects using nano-networks. It provides access to data that had previously been impossible to sense. This technology transition involving IoE is a concept called the Internet of Nano things [11].

With more relevant connections than machine-to-machine communications, IoE has enabled the global democratization of skills, including person-to-machine and person-to-person connections [12,13].

Accordingly, Auger et al. [4] argue that IoE extends the concept of IoT by exceeding the connections of things and integrating common impacts, risks, and economic benefits for the novel interconnected society. Thus, “intelligent services”, together with the “things”, represent the “everything” in IoE [14].

For Raj and Prakash [13], IoE is a superset of IoT and requires advanced capabilities within the area of information sharing. The IoE paradigm can extract and analyze real-time data collected from diverse and heterogeneous IoE environments, from simple sensors and actuators to complex robotic devices, and from autonomous service agents to human actors [3]. Thus, IoE applications require appropriate measures to be taken in the initial phases of their design and implementation [13]. Artificial intelligence (AI) integrated into smart devices provides the increasing deployment of innovative and useful IoE-based applications, where people and things interact appropriately within a social context and multi-user environment [11].

Processes are the core of IoE; they represent network “connections” and real-time data/information flows [15] among IoE nodes [16]. The result is smartness and intelligence [17], and real-time insights working in concert [18], far beyond IoT context disruptions [19], addressing the societal and organizational needs for more data and more actionable intelligence.

Actions and interactions within the IoE environment create and expand knowledge in a transcending process through which entities (people, things, and data) acquire new knowledge and new interactions are created in knowledge-creation cycles [20]. This transformation from data to knowledge in IoE provides essential insights and various possible applications [21,22].

Value-generating activities come through knowledge processes that filter data, information, and knowledge into a decision context, in which it becomes actionable intelligence [23]. A knowledge-based strategy for selecting and managing technologies and decision support artifacts (big data, data, information, knowledge, and intelligence) assists in the management and governance of data and technologies to ensure great benefit from IoE’s capacity to provide enhanced intelligent services.

**Figure 1:**
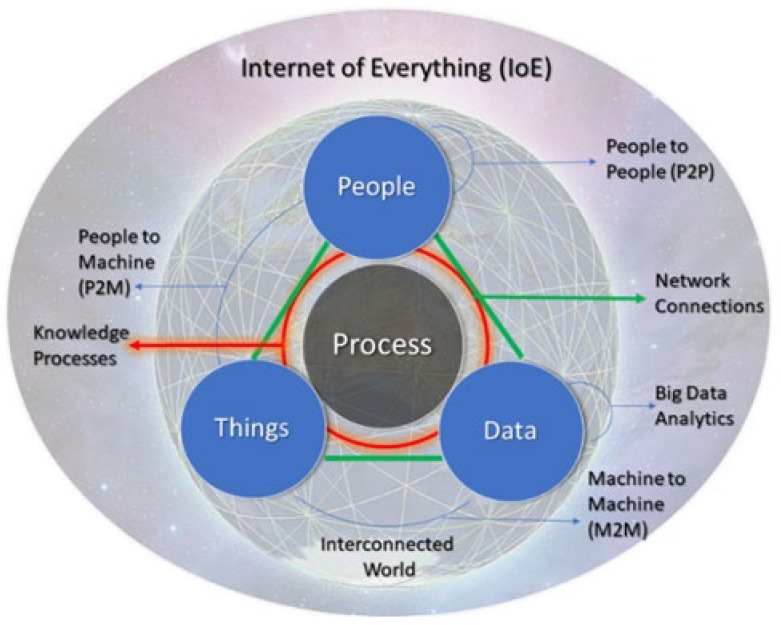
Internet of everything, adapted from [16].

Figure 1 shows the “four enablers” (people, data, processes, and things) in IoE.

Several taxonomies for IoT [24,25,26,27,28,29,30] and IoE [6,13,15,17,31,32], have been proposed. However, there are challenges concerning the ranking and managing of knowledge processes in IoE applications.

First, there is still a fragmented framework: (1) A lack of consensus and new demands are unique to the IoE context (e.g., empowering people and providing intelligence services and insights through the collaboration of IoE enablers [sensors and actuators]); and (2) A lack of consideration for the integration of IoE connections (machine-to-machine, people-to-people, and people to machine) from perspectives that support the high heterogeneity of existing IoE devices and the expected value creation in IoE applications—the perspective of knowledge that refers to actions, comprehension, and meaning derived from the information inside a context; the perspective of sensors and actuators physical characteristics and usage in IoE context, the perspective of information observation within ever-changing IoE contexts, and the perspective of infrastructure capabilities and resources required.

The successful adoption of a particular technology depends on the comprehension of its use and features [33]. Research on knowledge management has focused on understanding the complex relationships between data, information, and knowledge creation, and how they are impacted and benefited by the sources (or spaces) of data and information and the contexts in which they are analyzed and shared [34]. Therefore, an in-depth classification of IoE enablers (sensors and actuators) identifies issues and challenges in existing IoE applications regarding insights about managing knowledge processes that create value from the IoT context.

In this current study, we present research challenges in the IoE paradigm and a way forward in the classification of IoE knowledge enablers (sensors and actuators) to support the identification of critical knowledge sources that lead to actionable intelligence in IoE applications. We conducted a systematic literature review of existing IoE and IoT taxonomies. From this, we were able to present a knowledge-based IoE taxonomy that provides a consistent picture of IoE systems and their constituents (i.e., IoE sensors and actuators characterized in knowledge processes, observations, and network characteristics). The proposed taxonomy is based on knowledge in IoE applications—the knowledge provided or used by different types of sensors and actuators. The focus is to identify to what extent tacit knowledge from people, implicit knowledge generated from data analysis and integrated into smart devices, and explicit knowledge available from diverse data sources are composed to support observations that contribute to intelligent services in the IoE context. We then validated the defined taxonomy with 50 IoE applications in order to prove its quality attributes and identify research challenges.

The remainder of this paper is organized as follows: Section 2 introduces related works, including the theoretical background and state of the art of IoE theory; Section 3 contains the systematic literature review and methodology that provided the basis for the development of our proposed taxonomy; Section 4 describes our novel IoE taxonomy in detail; Section 5 discusses the qualitative outcomes; Section 6 presents the validation of the proposed taxonomy in diverse IoE domains; and, finally, Section 7 concludes the review with proposed directions for future research.

## 2. Related Work

Several authors have proposed taxonomies for dealing with IoE and IoT systems in the following distinct approaches: technology and architecture design [24,35,36,37,38,39,40], sensors’ capabilities [26,41,42,43,44,45], and observation context issues [25,27,28,46,47,48,49,50,51]. However, still, due to the considerable heterogeneity of actual IoT devices, these taxonomies have approach limitations, mostly restricted to enabling technology and infrastructure. Nearly all disregard the collective intelligence of IoE applications, in which human sensors are knowledge producers and consumers [52]. This human-thinking perspective integrated into IoE is still a research gap. Due to a large amount of heterogeneous and distributed IoE entities (human and non-human sensors/actuators), IoE requires the orchestration of intelligence in network connections concerning the knowledge provided, type of IoE entities, observation characteristics, and technological capabilities. This section provides an overview of related works, theoretical background, and state of the art of IoE theory.

### 2.1. Technology

As IoT is the technology architecture facet of IoE, many studies have focused on central concepts and their relationships in an IoT domain [35]. IoT is a self-configuring, adaptive, complex network with standard communication protocols that connects “things” to the Internet [24]. Yaqoob et al. [25] proposed an end-to-end view taxonomy to categorize and classify IoT architectures, considering requisites such as application domains, business objectives, enabling technologies, architectural requirements, network topologies, and IoT platform architecture characteristics. Recent studies have addressed different research challenges in IoT areas. In Mountrouidou et al. [26], the authors characterized IoT based on generic building blocks or primitives, defining IoT devices as sensing or actuating devices that can communicate with other devices and perform specific functions. Shahid and Aneja [41] proposed an IoT taxonomy, developing technologies and solutions for enabling IoT vision, which is related to smart objects’ ability to communicate and interact, either in building networks of connected items or with end-users or other entities in the network. Noura et al. [27] developed a taxonomy for IoT devices, networks and platforms related to the heterogeneity challenges of syntactic and semantic interoperability. Obinikpo and Kantarci [42] presented a taxonomy of methodologies to categorize types of sensors and sensing data. Other works have proposed taxonomies to classify the IoT’s connected objects, devices, and smart objects [43,44,45].

### 2.2. Management and Security

Management solutions must show grounds for efficient control of IoT systems. Sinche et al. [30] proposed a taxonomy related to IoT device management, and Perera et al. [36] surveyed a wide selection of techniques, methods, models, and solutions related to context awareness in IoT. Some authors have proposed taxonomies for security approaches in the adoption of IoT technologies and applications [46]. Using autonomic terminology, Ashraf and Habaebi [53] proposed a taxonomy that aims to group IoT security vulnerabilities and their mitigation solutions. Haron et al. [37] proposed a taxonomy of trustworthiness for IoT sensor data. Based on the notion of trust, semantics, Kotis et al. [54] presented an effective modeling approach towards supporting IoT entities’ selection and deployment. Alsamani and Lahza [55] studied the relationship between object characteristics, security, and privacy, and they proposed a taxonomy to categorize potential security threats in IoT. In Zhang et al. [56], the authors presented an extensive analysis of data security and privacy threats, protection technologies, and security solutions for edge computing. Oteafy and Hassanein [57] proposed a taxonomy of edge-IoT systems designed for rapid data acquisition.

Other studies have focused on network architecture for IoT and IoE. Thota et al. [58] studied the emerging communication techniques for the implementation of IoE applications. Gluhak et al. [38] provided a taxonomy for the scope and architecture of testbeds in the IoT. Naha et al. [59] proposed a taxonomy considering the requirements of the fog computing paradigm. In Hassan et al. [60], a taxonomy of edge computing classifies and categorizes existing edge computing paradigms for IoT. Ahad et al. [61] provided a state-of-art review of 5G- and IoT-enabled smart healthcare. Bellavista and Berrocal [39] presented a unified architectural model and proposed a new taxonomy after comparing solutions that had emerged for supporting the requirements of IoT applications. However, these works ignored the critical role of data flow throughout sensors and actuators of different types, how they collaborate to create value in cyberspace, and the context of observations.

### 2.3. Collaboration

Some works have focused on establishing an effective collaboration process between smart devices integrating humans in the loop [62]. A comprehensive look at IoT environment collaboration is presented in [28], in taxonomy to clarify how IoT enables collaboration. People (as customers) and applications are perspectives that nurtured the IoT taxonomy presented by Smutný [29]. Sholla et al. [47] argue that integrating socio-cultural and ethical aspects within a smart city architecture turns it into a people-friendly environment.

Hui and Sherratt [63] discussed how to strengthen human senses and capture human reactions, and they proposed a new taxonomy for disappearing user interfaces. Yebda et al. [64] reviewed existing solutions for social sensing. In [65], the authors presented a taxonomy based on the critical issues in mobile crowdsourcing. Chaochaisit et al. [66] presented an ontology for human localization sensors to address challenges in searching for users’ location-aware sensors. Sethi and Sarangi [48] proposed a novel taxonomy for IoT technologies and profiles and some applications that have the readiness to make a remarkable difference in human life. Salim and Haque [67] proposed a taxonomy for categorizing and characterizing urban computing technologies and also discussed the level of participation these technologies stimulate in modern society. In [49], the authors proposed a technical taxonomy for service composition in the IoT environment, based on functional and non-functional aspects. Bugeja et al. [50] proposed a classification model based on the functionality of smart home devices. Oberländer et al. [51] contributed to the IoT’s descriptive knowledge and presented a classification of business-to-things interactions to facilitate sense-making and theory-led design. In summary, these studies focused on the collaboration perspective but seldom investigated how smart sensors and humans contribute to knowledge creation to achieve a common goal.

### 2.4. Data Analysis

Many studies have focused on information flow, ranking information, and quality of data for the data-driven perspective and analytics applications. Bisdikian et al. [68] presented a framework for categorizing information products based on their value of information attributes. Different works have paved the way for effective utilization of the available opportunities in big data analytics and IoT. Shah et al. [69] created a thematic taxonomy for deploying these solutions collaboratively to provide guidelines for harvesting, transmitting, managing, and analyzing disaster data from diverse data sources in order to deliver valuable information to assist disaster management environments.

Focusing on successfully understanding and extracting value and insights from data analysis, [70,71], and [72] proposed semantic web techniques for better representation and exploration of sensor data. Qanbari et al. [71] incorporated semantic and linked data technologies to increase data quality. In [72], Rozsa et al. presented a taxonomy that identifies and categorizes sensors as the source devices to support publishing, discovery, sharing, reuse, and integration of sensed data.

Marjani et al. [40] explained the interdependence between big data analytics and IoT and proposed a novel architecture for IoT big data analytics. The authors in [73] surveyed the domain of big data by examining the different techniques utilized for processing and analytics. Another taxonomy on big data sensing and services was presented by Gao et al. [74]. The latest developments in the big data sensing field applied to context-aware big data systems were discussed by Subbu and Vasilakos [75], who proposed a taxonomy of recent works based on sensing platforms.

Ge et al. [76] surveyed big data technologies that stimulate knowledge sharing across IoT domains, whereas Moustaka et al. [77] proposed a taxonomy to integrate data science and smart city domains by focusing on concepts correlated to community data sources and analytics approaches regarding data harvesting and data-mining processes.

However, few studies have investigated the interaction between human-based sensors and smart devices and how and to what degree they contribute to and benefit from big data analysis in cyberspace in the context of knowledge creation.

### 2.5. Interoperability

Many studies have focused on the interaction among sensors and actuators in tri-space (cyber, physical, and cyber-physical). Kotis and Katasonov [78] presented a framework for supporting semantic interoperability between many distributed and heterogeneous IoT entities (sensors, actuators, and applications). Agarwal et al. [79] highlighted several core concepts from various mainstream ontologies and taxonomies, and they proposed an ontology for reusing and interconnecting existing ontologies. Alkhabbas et al. [24] proposed a characterization of IoT systems, which provides a holistic view of IoT systems synthesized from other existing taxonomies. Different works have discussed challenges in mobility and localization. Shit et al. [80] proposed a hierarchical taxonomy of the localization technique based on offline localization training, namely self-determining and training-dependent approaches. Saad et al. [81] presented a taxonomy that classifies variant localization algorithms. Pozza et al. [82] made a classification between mobility-agnostic and mobility-aware discovery protocols. Berger et al. [83] developed a multilayer taxonomy of digital technologies that comprises eight structured dimensions coupled with four layers of standard modular architectures: service, content, network, and devices.

Despite this, few works have investigated who participates in a smart environment and how things interact with human sensors through knowledge processes that lead to actionable intelligence. The critical goal of integrating human actors is to develop proper interfaces based on application domains, type of operation to be performed, and integration between human sensors within the role system [84]. As in a collaborative workspace [85], humans can maintain situation awareness in order to work collaboratively with smart devices. Moreover, things apprehend the situation, understand people’s requirements to enhance the value chain autonomously, and support intelligence services [86].

### 2.6. Challenges

Even though recent works are similar to ours in terms of coverage and analysis of the IoE paradigm, some approaches address our review criteria only to a varying degree. To clarify the contributions of the current paper, Section 5 summarizes a brief comparison of the scope of the proposed IoE taxonomy and 76 IoE and IoT previous works, selected in the literature review (presented in Section 3). The scope of the proposed knowledge-based IoE taxonomy is considerably more embracing in terms of visibility of intelligent connections between sensors and actuators in IoE applications than existing works. Some works touch upon knowledge creation and collaboration among IoE devices, while others propose taxonomies concerning specific areas (e.g., observations, infrastructure, sensor type, and analytics for IoT and IoE). They identify design challenges from several perspectives; however, they fall short in the two respects detailed below.

First, they do not explicitly address the characteristics of knowledge enablers (sensors and actuators) and to what degree they collaborate to improve efficiency in IoE solutions. In general, the identification of knowledge sources in human and non-human sensor nodes requires further research to enhance intelligence services through managing knowledge processes. Furthermore, for knowledge-intensive IoE applications, the governance of knowledge sharing in human–machine relationships are still mostly inadequate. This situation requires a complete taxonomy that leverages awareness from the length and breadth of the knowledge hierarchy, considering knowledge interaction and transformations from sensor platforms in collecting raw data for the foresight and intelligence that drive decision-making processes and provide outcomes and wisdom.

Second, although they provide an overview of hardware and software components in IoE systems, the studies do not categorize and organize them in a concise manner that provides a contextual understanding of the complexity of IoE enablers. The related works do not categorize the sensors and actuators in terms of knowledge users and providers and how they participate in knowledge creation in IoE applications. We propose a comprehensive knowledge-based IoE taxonomy that optimizes technological and architectural components as integrated resources that drive knowledge creation.

Providing a broad and forward-looking view of the IoE paradigm is, essentially, the principal contribution of this present study. This paper proposes an IoE taxonomy, based on knowledge, to elicit how intrinsic knowledge in sensors and actuators—in conjunction with enabling technologies and infrastructures—are applied in observations to produce intelligent services in the IoE context.

## 3. Research Methodology

Taxonomies are interpretations of reality and represent sense-making structures [87] (p. 51) for organizing information and knowledge into hierarchical relationships between the terms. This involves uncovering how the theories evolve, which enables researchers to study the essences and their relationships in the research territory [88].

As a form of classification [88], a taxonomy for IoE sensors that considers knowledge enablers elucidates how some types of sensors are pooled and used in diverse application domains and how issues with capabilities and observations can affect the quality of services and knowledge creation. In order to develop a taxonomy to guide the identification of critical knowledge in IoE applications and an in-depth classification of IoE enablers (sensors and actuators), we surveyed existing taxonomies related to IoT and IoE. The first step in developing our taxonomy was to review the existing classification schemes, semantic descriptions, and taxonomies, which could suggest design implications for IoE systems.

The methodological guidelines suggested by Kitchenham and Charters [89] for literature reviews guided this survey. Our review included contributions from the ACM Digital Library, IEEE Digital Library, ISI Web of Science, Science@Direct, and Scopus databases, which we considered to be the most relevant for finding specific studies in journal and conference papers in English. The following specific search string was sought: (“Internet of everything” OR “IoE” OR “Internet of things” OR “IoT”) AND (“taxonomy”) in the “Title”, “Abstract”, or “Keywords” fields.

We designed the search string to retrieve from the databases as many studies as possible that were relevant to the review, even if the query results returned articles not relevant to the survey. Relevant studies not retrieved after the first query were also included in a second iteration analysis in June 2020, considering studies likely to be explicitly related to IoE. Furthermore, most contributions were survey papers for IoT, which indicates a lack of maturity in work in the field of IoE.

We selected only studies published in English in journals (already published and in press), conference proceedings, books, and technical reports. After discarding the duplicates, a total of 394 candidate articles remained from the initial search (Table 1).

Each candidate article was subjected to the following series of steps before its eventual selection: (1) evaluate the title and read the summary; (2) retrieve the selected papers and read the introduction and conclusions; and (3) critically assess the contribution considering the degree of adherence to IoE applications and the contribution’s relevance.

Finally, after applying the filters, 76 articles relevant to this literature review remained. The studies were diverse and promoted different approaches. From the list of papers selected, it was possible to extract works related to IoT and IoE taxonomies, thus revealing the proposed IoE taxonomy. A qualitative analysis of the results summarized the main findings and provided some guidelines and a comprehensive overview of the topic that supported the novel knowledge-based IoE taxonomy proposal.

## 4. Proposed IoE Taxonomy

This section presents the proposed IoE taxonomy. For the conception of this taxonomy, we selected a method proposed by Nickerson et al. [88] for taxonomy development that has been adequately addressed for taxonomy development in the information systems (IS) domain. The proposed taxonomy identifies and categorizes sensors, attributes, and characteristics that are essential for developing IoE applications. This study is the first attempt to represent the types of knowledge (from sensors and actuators) in the IoE domain and how knowledge processes lead to intelligent services in IoE applications.

The development of an IoE taxonomy involves determining the characteristics of the sensors in IoE applications that arise from a refinement process at various stages to sufficiently satisfy the following qualitative attributes from Nickerson et al. [88] regarding the taxonomy:

1. Concise: has a limited number of dimensions and characteristics, restricted to what is relevant and understandable;
2. Robust: contains suitable dimensions and characteristics to distinguish the objects of interest;
3. Comprehensive: includes appropriate and enough dimensions to classify all known objects within the domain under regard;
4. Extendable: allows for the insertion of additional dimensions and characteristics within a size to contemplate new incorporated objects;
5. Explanatory: provides useful explanations and valuable descriptions of the nature of the objects under study.

Additionally, developing a useful taxonomy is a search process of design [88]. Kotis et al. [90] presented requirements for a well-defined collaborative and iterative methodology, addressing practical aspects that drive consensus on developments—a “live” method of development in which the artifacts evolve over time. An artifact must preserve its liveness, evolution, and reusability during its life cycle (i.e., it may be in-use in a particular time or instant, be under constant maintenance or update and be used in applications/projects beyond its original purpose).

We followed an iterative method during the development process, as suggested by Nickerson et al. [88], and a conceptual to the empirical approach, based on the surveyed existing taxonomies related to IoT and IoE (Section 3). Furthermore, a collaborative approach that relied on authors´ insight, experience, intuition led to proper identification of the proposed dimensions and characteristics, as resumed in the following stages of the development.

For Nickerson et al. [88], the taxonomy’s purpose (meta-characteristic) drives the taxonomy’s dimensions and characteristics. Each element or classification proposed in the taxonomy should be a logical outcome of the meta-characteristic. Our aim was “to guide the identification of critical knowledge in IoE applications, an in-depth classification of IoE enablers (sensors and actuators) based on the knowledge they provide in intelligent tasks”.

The development process ended when both objective and subjective conditions have been met [88]. During the iterations processes, new characteristics were identified and included, and when any characteristic turned out not to be relevant, they were eliminated after consensus. Further analysis succeeded until reach the ending conditions, engaging authors in close collaboration towards shaping commonly agreed dimensions and characteristics. We used two objectives ending conditions: no new dimensions were added in the last iteration, and every characteristic was unique within its dimension. Subjectively, the process ended when the taxonomy was determined to be concise, robust, comprehensive, extendible, and explanatory [88] and fulfilled the quality requirements of liveness, evolution, and reusability [90] that are suitable to dynamics of the IoE pervasive environment.

Accordingly, ranking knowledge in IoE sensors is a matter of eliciting the main characteristics in IoE applications. In order to understand the IoE domain, we applied specific questions by answering the 4 ws (what, when, who, and where) and 1 h (how) identified using the 4W1H methodology [91,92]. This methodology addresses the challenge imposed due to the high heterogeneity of existing IoE devices. A similar approach was proposed in [68] to measure the quality and value of information when considering the value created by the IoE in applications. These questions guided the definition of the following four complementary categories that drive the purpose of taxonomy dimensions and characteristics:

(a) Knowledge: regarding knowledge in action; that is, the artifact or information inside a context (*what*) with comprehension and meaning;
(b) Type: typifies sensors and actuators—*who* they are, their physical characteristics, their usage, and their role in IoE context: sensors or actuators in cyber, physical, or cyber-physical presentation;
(c) Observation: the physical context in time (*when*) and space (*where*); that is, the instant and location that the information content was sensed or perceived within ever-changing IoE contexts;
(d) Capabilities: *how* the information is flowing, the infrastructure capabilities, and the resources required.

**Figure 2:**
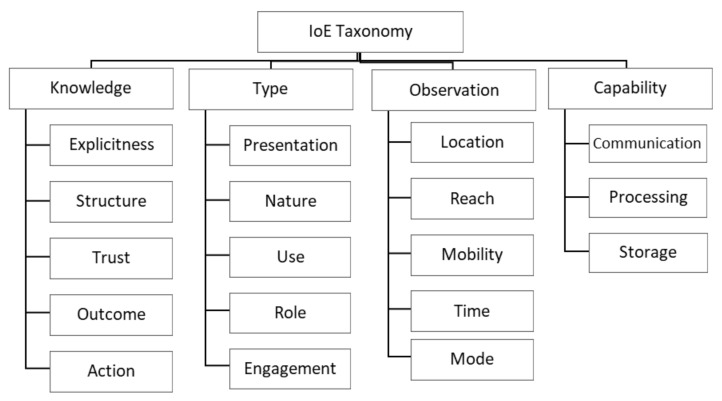
IoE taxonomy.

For this stage of development, we used the top-down development process, starting with defining the most general categories (*knowledge, type, observation,* and *capabilities*). We then selected dimensions and characteristics previously derived from a theoretical foundation from reviewing the related literature, as presented in Section 3 and grouped them in related *knowledge, type, observation,* and *capabilities* categories, revealing the resulting taxonomy. Our IoE taxonomy consists of four categories (see Figure 2) and groups 18 dimensions, each comprising of mutually exclusive and generally collectively exhaustive characteristics. Section 4.1 describes the *knowledge* category, which analyses the knowledge characteristics and the value created by IoE applications. Section 4.2 details sensor characteristics related to their use in IoE applications (*types*). Section 4.3 presents the *observation* category, which classifies how data are sensed and gathered in IoE observations. Finally, in Section 4.4, the sensors’ *capabilities* are classified into a few dimensions that address the technological aspects for designing IoE applications.

### 4.1. Knowledge

**Figure 3:**
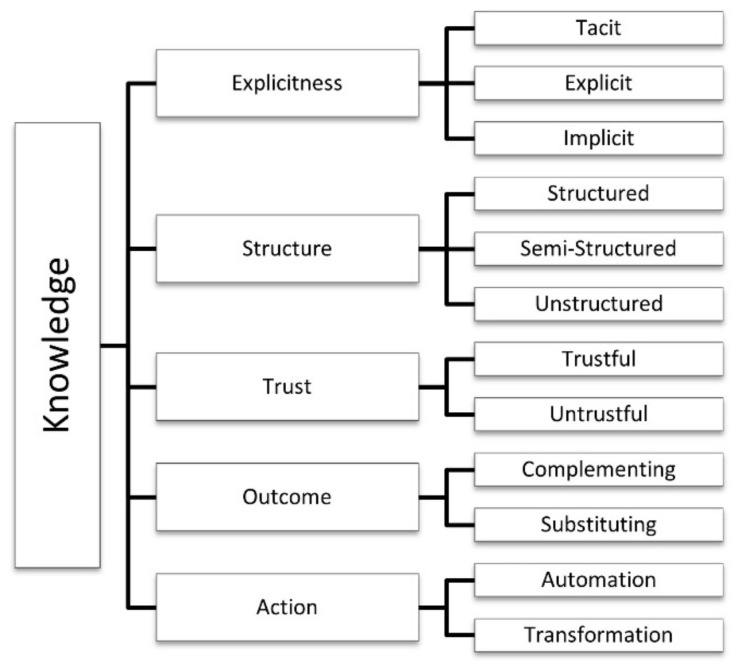
IoE Taxonomy: knowledge category with dimensions and characteristics.

The *knowledge* category contains five dimensions related to knowledge creation and information flow: explicitness, structure, trust, outcome, and action. Each has its own specific sub-dimensions or characteristics, as shown in Figure 3.

#### 4.1.1. Explicitness

IoE environment architectures consist of IoT standard architecture [93], but with the addition of the human element (which acts as a node) and intelligent services to the IoT network [32]. knowledge discovery approaches used in developing IoT solutions [82], which involve sharing information from smart objects, should be optimized by examining how humans process data sources of information to form knowledge [41]. For Perera et al. [36], this requires knowledge from different perspectives, for example, knowledge of sensors, applications, users, and so forth. Moreover, these uncovered knowledge patterns are analyzed and integrated for subsequent use in real time, using multiple knowledge management approaches [76,94,95]. The intelligence of connected things varies from non-existent to absolutely rational [24]. There are different kinds of knowledge, and it demands distinct representations. A taxonomy is a central link between knowledge engineering and knowledge management [96]. Regarding explicitness, this work classifies knowledge provided by sensors in IoE applications into three distinct types:

- Tacit: This knowledge is rooted in actions, experiences, and involvement in specific contexts. Tacit knowledge consists of people’s knowledge based on intuitive evaluations of sensory inputs and perceptions, which is sometimes hard to express (i.e., feelings, beliefs, insights, values, and ideals) [97]. The increase of human senses through sensor and data fusion and context awareness is the essence that supports smarter wearable devices for relating mutually with human cognitive memories [98].
- Explicit: This knowledge is codified and articulated knowledge (i.e., the form of knowledge that is easy to codify using formal language, procedures or principles) [97]. Explicit knowledge from hard sensing-based data acquisition results in discovering hidden patterns in the aggregated sensor data [42,66]. The explicitness denotes awareness of a fact or artifact, which means the application of knowledge [98] from efficient scheduling of the resources in IoE applications [82,99]. Sensors continuously generate enormous amounts of data, with the value created being conditioned to its analysis.
- Implicit: Knowledge is not explicitly represented in the knowledge base but is inferred from it by using several assumptions [100]. Thus, implicit knowledge may be implicit information intertwined in information systems and data sources [97]. Myriad data analytic algorithms can be executed to extract a higher level of information from sensed data [99]. The value created by implicit knowledge emerges from machine learning and AI technologies, mainly in machine intelligence services [101]. It consists of outputs to make predictions oriented toward decision support and automation in diverse IoE application scenarios [102].

#### 4.1.2. Structure

The combination of data streams with background knowledge enables meaningful analysis to derive higher levels of abstraction and deliver quality actionable information to IoE services [71,95,99]. Sensor data are a piece of explicit knowledge with metadata characterizing the body of evidence [68]. The distinctions between data, information, and knowledge are largely irrelevant [97]. Knowledge is created by transforming the multiple data formats collected (structured, semi-structured, and unstructured) [103] into high-level information [36,64,94,104], and useful knowledge patterns [36]. Descriptions of these data formats are given below:

- Structured: These data have an identified format and a relational structure, frequently accessed using a standard SQL-type language and stored in relational database management systems. Typical examples of structured data are string, numeral, and date. [105].
- Semi-structured: These data cannot be managed by conventional database management system techniques, but the interpretation and analysis of these data require comprehensive and intelligent rules. Typical examples of semi-structured data are extensible markup language (XML) and JavaScript object notation (JSON) data. [50,101,105].
- Unstructured: These data do not follow any specific format and are often represented in a rather complex structure that contains hidden relationships. Examples of unstructured data are videos, text, time information, and geographic location [40]. With the amount of data generated by sensors, devices constantly produce large volumes of structured, unstructured, and semi-structured data, which results in ”big data” [73,74].

IoT processing of sensing data streams provides ubiquitous sensing services [42,102,106]. Data aggregation processes are vital for improving the quality of the designed system [107]. Big data technologies assist in data processing [76], the uncovering of new and valuable insights and information from incorporated data sources [28,69], and in improving prediction and decision-making [102].

#### 4.1.3. Trust

In a hybrid human-based and device-based environment, such as IoE, data’s trustworthiness can be estimated mostly by the sensor nodes’ reputation [37]. Trust management is a decisive challenge for data access and data storage on IoE applications [49,108].

Dynamic and heterogeneous network environments and the diversity of devices connected in the IoT generate an extensive array of potential security threats [27,60,61,109]. The network interoperability level should address concerns such as the security of the data to be transmitted [64], and a coherent IoT architecture would provide a layer of data security [110,111] since the IoT has no uniform architecture. Approaches and methods to improve users’ awareness about the effects of potential IoT threats may mitigate the risk of exposure [53,65,68,112].

Knowledge assets vary in veracity levels [97], between the extremes of truth and untruth [52]. In some broad sense, the value of knowledge depends on the quality of the sensors’ information. Security approaches must be made self-sufficient and autonomic, with the minimal manual human intervention [53]. Sensor networks’ applications need support regarding privacy, security accuracy, timeliness, relevance, completeness, and provenance [46,68]. The data source’s reputation represents the source’s truthfulness in providing quality content to handle changing external requirements and contexts [101]. Any direct or indirect connections of user information with connected objects within IoT landscapes categorize trust in communication and security issues [108] [110]. The trust values are considered based on the reliability of devices and the level of security and trust engaged in implementing and operating the connectivity [113,114]. Knowledge of sensors and sensor data in IoE applications is either trustful or untrustful:

- Trustful: Based on protecting both user and service provider privacy precedents [40]. Constituting meaningful identity, using trusted communication paths, and preserving contextual information is essential to guarantee the protection of users’ privacy in the IoE environment [115]. The work in [55] addressed the security of IoT objects and privacy issues by merging identification, authentication, and authorization into one argument: access control. The security dimension encompasses five concepts: access control, confidentiality, integrity, availability, and non-repudiation. Different studies have covered concerns such as anonymity, liability, and moral, ethical, legal, cultural, and regional parameters, among other things [39,45,47,116].
- Untrustful: False or misleading data culminates in wrong decisions and critical consequences and lead to uncertainty at all knowledge transformation levels. Incompleteness in data occurs at the lower layer of the sensor readings or raw data collected. Vagueness frequently appears at a higher level of contextual information [37,69]. Possible security risks associated with IoT data are the heterogeneity of the smart devices and the nature of sensed data or authentication among different trust domains [56], which further complicates access control decisions.

#### 4.1.4. Outcome

The IoE paradigm impacts human interaction with everyday objects. Considering the type of information exchanged between humans and the system [84], the expected outcomes from IoE applications provide multiple tiers of cognition with the fine-tuning sensory acquisition from heterogeneous contexts [57]. Distinct levels of collaboration between IoE resources require efficient solutions. Human sensors peculiarities contemplated by collaboration theory and technical aspects of user interaction are challenges in computer network theory [28]. It is imperative to provide awareness of collective intelligence and where the intelligence is [113], representing the outcomes expected in designing the IoE solutions, based on the application domain [28,104].

The outcome dimension refers to the degree to which knowledge sources (things and humans) contribute to knowledge creation in IoE intelligent services. Relevant knowledge contributions from human or non-human enablers (sensors or actuators) either complement or substitute (or both in some cases) to provide improved outcomes reached through knowledge sharing processes, and sometimes automating or transforming traditional tasks [55] into IoE environment disruptions:

- Complementing: Represents knowledge sharing between IoE sensors and actuators. Complementing outcomes occurs when humans utilize mobile devices like sensors to collect their observations and information about the environment and infrastructures [25,51,65] or when artificial intelligence complements human knowledge.
- Substituting: Provides insights and novel interpretation of reality to enhance the quality of life (livability), regarding knowledge acquisition as the “core element” and the realization of “intelligence” [77].

#### 4.1.5. Action

The Action dimension refers to knowledge creation. Actionable intelligence is meaningful for humans to promote automated processes [51], ranging from creating value when used in a specific usage context [25,68,117] to transforming and changing the state of their environment [24]. Big data analytics aims to improve the understanding of data, thereby supporting useful and timely decision-making with the refined information gathered [40,42,69].

The goals of IoT systems range between general and specific and include monitoring, reducing costs, and improving processes [109].

For Russell et al. [117], even in the case of uncertainty, a rational agent is one that acts to achieve the best outcome or the best-expected outcome. There is a close interrelationship between intelligence and automation [55], or creating and pursuing goals through transformation. Sensor information in IoE applications provides either automation or transformation of the IoE environment, which are defined as follows:

- Automation: the aptitude to make cognitive decisions related to a given situation, which guarantees the right action is performed. The automation of tasks and dependency on machines may reduce human abilities [105]. When combined with AI and machine learning, new applications will benefit from automated decision-making [106], with efficient usage of network resources, minimization of operational costs, coordination of computational resources, and efficient and effective data management mechanisms [60] associated with the quality of experience [104,118].
- Transformation: an enormous number of raw observations (created by the machine and human sensors) can be transformed into higher-level abstractions [57] that are meaningful for human or automated decision-making processes [55]. When an IoE solution provides transformation, smart things act independently, with minimal or no human intervention [51]. With the support of wireless communications and AI, humans benefit from improvements in technological advancements [42,101] by collecting, modeling, and reasoning the context [36].

Considering how actions generate changes in the environment to achieve the desired goal, automation and transformation processes may occur in the short or long term or may represent a prominent solution. Some works have explored the implications of the IoE for value creation and decision-making provided by smart things and big data [15,39]. However, our study is concerned with how humans respond and interact with the environment in assisting the evolutions of future systems (defined in [15]), which can be:

- Reactive: having the ability to promptly react to a changing environment;
- Adaptive: having the steadier ability to adapt their behavior to changes;
- Predictive: having the ability to use computation and analytics techniques to identify relevant patterns, in-depth knowledge of the environment, and the most appropriate solutions or possible evolutions to each IoE system situation.

### 4.2. Type

**Figure 4:**
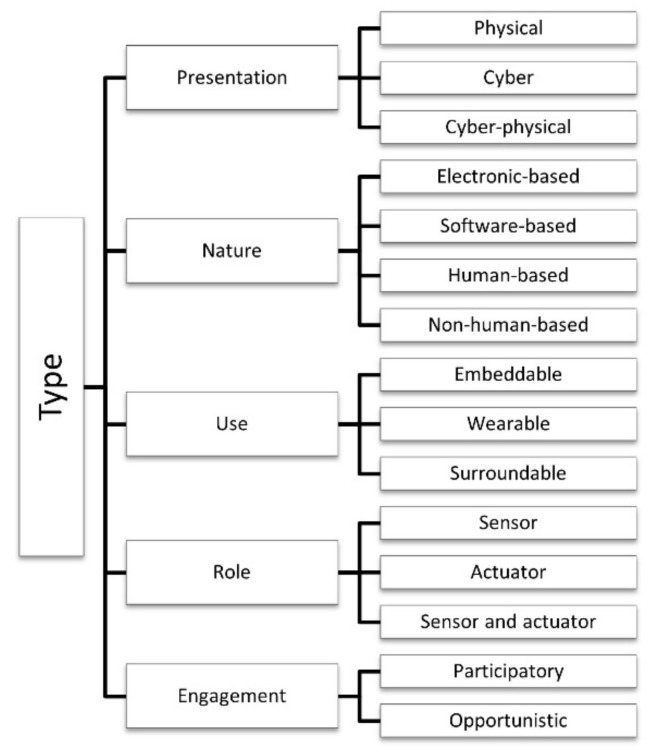
IoE Taxonomy: type category, its dimensions, and characteristics.

The *type* category contains five dimensions or subcategories for the classification of sensors and actuators: presentation, nature, use, role, and engagement. Figure 4 highlights the type category, its dimensions, and characteristics.

#### 4.2.1. Presentation

Presentation refers to the physical aspects of sensors and actuators that interact with the physical world. The physical and virtual world can be merged by integrating computation and physical processes in one of the following ways: a) physical, b) cyber or virtual, and c) cyber-physical or logical [30,46,62,67,79,84].

Humans are content receivers and can act as a sensor collecting data for the sensory systems or actuators performing actions, but humans are also content providers who share diverse and relevant types of spatial-temporal data [59,63,65]. The physical dimension characterizes the mobility of the system’s things and the dependency of the collaboration of human and non-human devices [28,39]. Accordingly, sensors and actuators can be classified as follows:

- Physical: Physical entities are tangible devices that generate sensor data or perform actions changing the environment. The data retrieved from physical sensors represent a low-level context [36]. Examples of physical sensors are temperature sensors, pressure sensors, biosensors, light sensors [6], and human sensors [35]. Examples of the physical actuator are a door opener actuator invoked by an intelligent system and human actuators.
- Cyber or virtual: An abstract information entity that invokes sensor or actuator functions but does not directly interact with the physical world. Examples of cyber or virtual entities are computer programs and systems, communication processes, and monitoring activities with no physical body (e.g., sensing web service) [51,66,74]. Virtual entities use web services technology to send and receive data from many sources [36].
- Cyber-physical or logical: Represents the connection of the cyber and physical worlds as a combination of physical and virtual entities to generate meaningful information [25,83]. Similar to virtual entities, they commonly use web services technology to send and receive data and interact with the physical world [36]. They are autonomous objects augmented with sensing, actuating, processing, storing capabilities [45]. Examples of cyber-physical entities are web services dedicated to providing weather information resulted from physical sensors that sense weather information and virtual sensors that process historic weather data.

#### 4.2.2. Nature

This dimension is related to sensor or actuator knowledge, intertwined with its architecture and functionality [43]. A sensor is anything that observes, and an actuator is anything that performs defined actions [119]. People can be modeled as sensors and actuators [117], so anything that acts individually to perform a task in the IoE context is an individual IoE device [59]. Knowing the nature of knowledge source devices is crucial for publication, discovery, sharing, reuse, and integration of information within the IoE environment [72]. Human beings with dedicated roles, as well as machines, devices, and services [35,106,110], implies system constraints when it interacts with the physical space [46]. Humans are content receivers through the sensory systems and also content providers—mainly through sensing and actuating abilities [63,120], and through tacit knowledge and experiences that can affect their actuations in IoE applications and cognitive tasks.

The level of autonomy of an IoE sensor or actuator (human or non-human) refers to its ability to act independently [24,83,113]. Several works have identified entities—sensors/actuators—types according to activities carried out in physical and virtual worlds [30,37,62].

According to their built-in nature, sensors and actuators in IoE are classified [62] as follows:

- Electronic-based: Define physical IoT devices constituted of electronic or mechanical systems that sense or actuate physical phenomena.
- Software-based: Define virtual entities that process information from data sources or generate analytical results.
- Human-based: Refers to humans or virtual entities based on knowledge provided or expressed by human perception about any phenomena arising in their physical, virtual, or social environment.
- Non-human-based: Define biotic sensors/actuators or virtual entities based on knowledge data provided by biotic perception about any phenomena arising in their physical environment. In the constantly growing area of animal cognition, sensor networks monitor the health and well-being of animals in livestock herds and in animal surveillance applications [121].

#### 4.2.3. Use

Refers to the physical characteristics of physical IoE sensors or actuators related to their usage in a particular application. The devices inherit the attributes of their owners or of the entities or places [79] to which or where they are attached [43,66,113]. A wide variety of objects—a group of infrastructures and devices [44] such as embedded devices, sensors, and actuators—have integrated communication and strong interactions to create a ubiquitous environment [71,106,110]. A taxonomy for IoT sensors communicates how distinct types of sensors are combined and used in specific application domains [72].

Smutný [29] described things according to how they are used or applied in relation to humans:

- Embeddable: Things that are in the user or under the user’s skin, that are non-autonomous, or embedded in carry-on devices [42]. The level of autonomy ranges from human-companion device tasks [65] to opportunistic devices, which decide and act independently [24,28]. For example, a mobile phone is a ubiquitous, convenient and user-friendly device and has many sensors embedded [48], which is why it has turned into a global mobile sensing device [67].
- Wearable: Things that rest on a person’s body or can be used, worn, or attached to their owners and enable accurate detection of the wearers’ motions [50,63,64,75].
- Surroundable: Things that are autonomous, near or around the user, but which have no physical contact with the user. Recently, several non-contact techniques have been interpreted as highly valuable in dealing with highly infectious diseases such as COVID-19. In a pandemic scenario, non-contact sensing was able to detect information without direct contact with the patients and without devices physically touching the body [122].

#### 4.2.4. Role

IoT devices have sensing and actuating capability according to defined rules under various scenarios [59,72]. They perform sensing and actuating functions [24,26,51] that help in interacting with the physical environment [48]. An IoE device or enabler can be a sensor, an actuator, or a sensor and actuator [44,77,106].

- Sensor: A device that observes and senses. Sensing is a read operation over a context entity. The data collected by a sensor is stored and processed intelligently to derive useful inferences and to support the decision-making process [46]. Sensors are monitor devices and physical entities, which provide the information required to immediately control actuators, whereas actuators act on the physical entity or control other things [28,35,114].
- Actuator: Affects a particular domain of the physical space or a combination of both. Actuation is a write operation over a context entity, in which the conceptual entity represents the domain of a sensor or an actuator [44]. Actuators perform the decided actions and effect a change in the environment [36,39,48].
- Sensor and actuator: This device is a hybrid of the two previous categories, and it can gather data and act within its environment.

Processing and analytics (fixed process or algorithm, machine learning, or AI) do not fit within this classification [113].

#### 4.2.5. Engagement

Participation is an interaction between people with existing technologies and occurs at different engagement levels [67]. Engagement refers to sensing tasks. In data acquisition, it can be both opportunistic and participatory, and it provides sensory information that collectively forms knowledge.

For example, enhancing human senses is possible when machines interact with humans or provide remote operation in perceived real time in ubiquitous computing [57,63]. Cooperative smart things can interact with other entities of the IoE in order to achieve a unified objective [15]. With mobile crowdsourcing, the primary information shared voluntarily is user knowledge and opinion, along with location as the only sensor information [65]. The engagement of a sensor node in an IoE application is one of the following:

- Participatory: The IoE enabler (sensor node or actuator) is actively involved and actively reports observations [120]. It can provide information about the environment or surroundings, as well as any other sensory information that could be on social groups (social sensing) or with everyone (public sensing) or at the community level [37,67,106].
- Opportunistic: The IoE node has minimal or no involvement—it senses and monitors tasks running in the background. Embedding sensors trigger the data automatically (either periodically or based on events).

### 4.3. Observation

**Figure 5:**
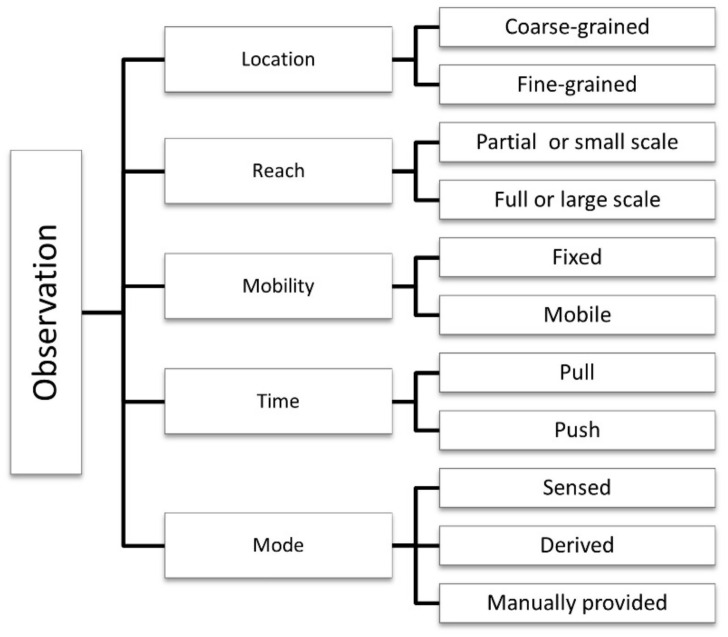
IoE taxonomy: observation category, its dimensions, and characteristics.

The *observation* category contains five dimensions or subcategories related to sensed context: location, reach, mobility, time, and mode. Figure 5 emphasizes the dimensions and their sub-dimensions or characteristics.

#### 4.3.1. Location

Location is used to describe the spatial context (physical context) of users/devices within a local or global network [24,113]. It represents the geophysical position of a sensor or actuator in absolute values, identifying the coordinates (latitude and longitude) or relative specifications through location tags [45], which is obtained manually or automatically [120,123]. It represents the definition of an area covered by a particular object [79]. Sensors that are randomly deployed get the required information about the target environment [81].

Location systems can be categorized as context-aware systems [75]. The precise location of an object is critical since location plays a critical role in context-aware computing [36,66,80]. Moreover, aggregation of knowledge patterns facilitates reduced data transfer in distant environments and minimizes bandwidth use [94]. Some physical measurement-based localization schemes are classified as coarse-grained and fine-grained [80].

#### 4.3.2. Reach

Reach classification distinguishes between individual and collective knowledge. It refers to an environment of sensing interest [71]. Sensors are becoming more sophisticated in technology advances, cheaper in price, and smaller in size. This evolution stimulates large-scale deployments [36], and dense geographical distribution [60].

The domain of interest represents the applicative domain in which the device is operative [79,81] and ensures that IoT services are accessible or reached only by authorized access [113,124].

The prevalence of mobile devices, such as smartphones, has triggered challenges for mobile networks worldwide [125], as well as novel classifications, such as collective knowledge classified into individual or group, internal or external, full or partial domains. For example, a conglomeration of sensor data stored on cloud infrastructure can be designed as big data sensing, and based on the reach of its sensing requests and requirements [74], it can be referred to as a) private, b) public, c) community, or d) hybrid big data sensing.

In crowdsourcing, regarding the boundaries of the individual scope in which crowds collaborators are immersed, the reach can be classified as ranging from small to large-scale (from a person to a group, community, city, and so forth) [28,65].

#### 4.3.3. Mobility

Mobility, which is also called monitoring continuity [36], is one of the main characteristics that enables identification of the state of sensors and actuators and their capability of movement [26,36,39,43,44,80], with significant implications on device operation, connectivity, and location management [30,48,80]. Devices are classified into two categories: static/immobile/fixed and mobile [26,77,82,113].

- Fixed/static/immobile: Objects that remain static to a specific location or cannot move. Their observations are restricted to a specific location, in a static or very constrained (in terms of mobility) environment that is not designed to move (relative to their point of installation) without being uninstalled.
- Mobile: The objects move [44], and their location may be calculated in absolute coordinates or relative to reference nodes in the network [81], requiring wireless communications to transmit data and allow configuration and control [113]. Their movement and mobility capability are controlled independently (or autonomously) or dependently through device users [43].

A self-moving device moves autonomously and relative to its setup/installation point, without being uninstalled (e.g., smart car), whereas a non-self-moving device does not move autonomously but can still move relative to its original location without being uninstalled [26,77].

Mobility of the things in the system is dependent on the collaboration of the items physically coupled with the humans in the system [28], as in crowdsensing applications, in which geographically dispersed users actively (participatory) or passively (opportunistic) collect data with their smartphones [51,75]. Classifications between mobility-agnostic and mobility-aware [82] highlight an approach that ignores knowledge about mobility and the ones that consider and exploit it for optimization [12].

Challenges related to mobility include frequent disconnections and handoffs, which affect perfect connectivity [126]. Mobility techniques in the cloud, fog, and edge architectures [60] support mobility, and other protocols apply routing and resource discovery mechanisms [39].

#### 4.3.4. Time

Time represents the instant of observation (i.e., timestamp) [79]. Information about time and location are critical features of some applications (called spatial-temporal-aware applications) that require tasks to make observations at a specific location during a defined period [120]. In [24], latency relates to the time an IoE system needs to answer to a stimulus. Interaction between IoE smart devices can influence the service’s response time to end-users. The time interval between the initiation and the conclusion of the task is the response time [49,107,109].

The time dimension depends on how sensors are requested or provide data to the system in specific periods or on an ad-hoc basis (as software system makes a request), which is characterized as the following two distinct methods that were proposed in [107]:

- Pull method: The software component in the control of obtaining sensor data from sensors makes a requisition periodically (after specific intervals) or instantly obtains sensed data [107].
- Push method: The physical or virtual sensor pushes data to the software component in the control of obtaining sensor data periodically [36,107]. In many cases, a sensor observation can be the result of a local sensor data fusion [68].

Real-time applications monitor the state of the environment and react to changes accordingly and in a timely manner.

The deployment of IoE applications in real-world scenarios creates a massive amount of data from real-time interactions, usually at high data rates. It faces challenges as temporal data consistency related to the coherency between the value of the data in the system and its environment state [107]; and high latency during interactions [39], when inferred contexts evolve with time [91,94], and the exchanged data may not be accurate.

Hard real-time data cannot accept any delay; in contrast, soft real-time data can accept various bounded delays. Delay-tolerant applications can be categorized as nonreal time [60,107]. In offline circumstances, the delayed transmission may be crucial to address quality and security constraints [37,65].

In real-time situations, timeliness [69,107] describes data processing in a specific deadline, which is real time, near real time, or batch processing [113].

- Real time: refers to the immediate data processing to provide instant results for a time-sensitive application.
- Near real time: refers to situations when the delay time is still relevant for the application, but the computation process is not as immediate as real time.
- Batch-processing: refers to situations when data are first collected and processed at a predetermined interval or when a specified volume of data is available [37].

#### 4.3.5. Mode

The combination of sensors serving different purposes and data generated in IoE applications implies the need to classify data sources and information in the IoT context [72]. During real-time data harvesting, it can be challenging to determine the possible relationships among heterogeneous knowledge sources [69]. Smart device sensors are either active or passive sensors, depending on their usage and functionalities. If the sensor data collected are reflected in the same way as designed, this is called active functionality. However, sensors operate passively when collected data are interpreted or processed in new ways [17,65].

Eris et al. [28] defined how much interaction is required within the network in three levels of collaboration interdependence [28]:

- Pooled interdependence: The lowest level of collaboration, in which each collaborator barely contributes to the collaboration environment and benefits from the contributions of others. The collaborators neither synchronize nor negotiate the nature of each other’s contributions.
- Sequential interdependence: The middle level, in which the contributions of one collaborator become the inputs to another collaborator contributions. In this case, there is a temporal ordering of the collaboration efforts.
- Reciprocal interdependence: The highest interdependence level, in which one collaborator’s contributions are the next collaborator’s inputs, and collaborators must also negotiate the nature of each other’s contributions to the collaboration environment.

The Mode dimension refers to the way of linking the physical and digital world in order to acquire context [127], and it can be either sensed, derived, or manually provided:

- Sensed: Data gathered through sensors.
- Derived: Includes the sensed data stored in databases or the information generated by performing computational operations on sensor data. Data aggregation is the ground for the application’s workflow and unconditionally impacts the application’s quality. Distinct aggregations may have specific requirements to be supported by design [107].
- Manually provided: Human sensors provide the context information [36].

### 4.4. Capabilities

**Figure 6:**
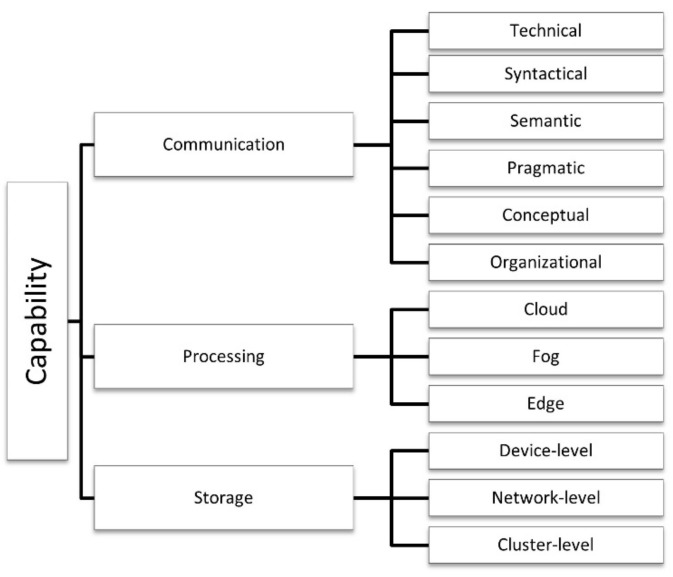
IoE Taxonomy: capability category, its dimensions, and characteristics.

The *capabilities* category contains three dimensions or subcategories (communication, processing, and storage) and refers to the processing power and storage capacity of the underlying technologies and communication protocols. Each dimension has its specific sub-dimensions or characteristics, as represented in Figure 6.

#### 4.4.1. Communication

The communication capability refers to the sensors’ ability to communicate and change information locally. This ability may vary at different levels of interoperability between IoE sensors and systems and be classified as no connection (no connectivity between enablers), technical (basic network connectivity), syntactical (basic interoperability and data exchange), semantic (understanding about the semantics of the data), pragmatic or dynamic (applicability of the information), conceptual (shared view of the pervasive world) [27], or organizational (coordination and alignment of business processes across organizational boundaries) [128]. Additionally, based on communication capabilities, IoT devices are classified into two categories: gateway devices and constrained devices [43,45]. Moreover, according to their abilities to interact with other objects, IoT objects can be classified into four levels (Level 0–Level 3). Level 0 objects only receive, and Level 1 objects only send information. Level 2 objects can perform both operations with one object, while Level 3 extends the interaction to any other object [50].

Different networking protocols and technologies provide networking interoperability in IoT [27,48,114]. IoT systems can exploit several types of networks with different characteristics in terms of size, data transfer, coverage, latency requirements, capacity, and supported reachability [69,74,75,83,110]. The central networking and communication technologies are local area networks, wireless local area networks, wireless personal area networks, wide area networks, metropolitan area networks, wireless regional area networks, body area networks, mobile communication networks, wireless metropolitan area networks, satellite networks (e.g., GPS) [24,118,129], Neul, IPv6 over low-power personal area networks (6LowPAN), low-range wireless area networks, cellular Sigfox, narrowband-IoT, and thread or mesh technologies such as Zigbee and SDNs [25,30,113].

There are three types of communication protocol that enable IoT to interconnect and communicate: (1) device-to-device, which is applied to communication between mobile phones within reach and is the next-generation of mobile networks; (2) device to the server, in which the sensed data are sent to the servers, nearby or away from devices (applies to cloud processing); and (3) server-to-server, in which servers transmit data between each other—mainly used for mobile networks [99].

#### 4.4.2. Processing

The sensors and devices used for data collection also vary in their processing capabilities [130]. The study of Mon et al. [127] classifies sensors as high-end or low-end devices, depending on resources and computational capabilities. Low-end devices are resource-constrained with regard to energy, processing power, and communication capacities. The processing capability refers to the sensors’ ability to process aggregated data locally [55].

For IoE systems, data are automatically processed to deduce knowledge and generate actionable insights. In general, data processing techniques are either historical or proactive. Historical data processing is related to knowledge discovery, whereas proactive data processing provides predictive and actionable insights [24]. A broad category of applications participates in the continuous generation and analysis of high-volume heterogeneous stream data. Next-generation applications will be developed to handle the data in streaming mode and on-the-fly as the value of data resides in its real-time processing [131].

Analytics technology refers to the systematic computational analysis of transforming a variety of data from different sources into information [105] and applying data fusion and mining techniques [94] to make intelligent decisions at the following distribution levels: (1) the device level, where devices are responsible for storage and computing process; (2) the network level, which demands remote communication to fog computing nodes (hubs, base stations, gateways, routers, and servers); and (3) cloud level, which demands remote communication within a group of interconnected servers [24,38,73,114,118].

Cloud, edge and fog computing are critical aspects of the centralized and decentralized IoE environment, considering that devices that have restricted compute and memory capacity need to delegate these functions [25,26,29]. Integrated with cloud computing, edge computing can efficiently address the processing problems related to edge big data. Since in the edge computing paradigm, the data are at the edge of the network [28,56].

A variety of cloud computing and edge computing paradigms are mobile cloud computing, mobile edge computing, and fog computing [32,60,126]. Cloudlets, mobile edge computing, and fog computing are edge computing technologies and rely on virtualization, while mobile cloud computing processes the data of mobile applications at a remote cloud data center.

#### 4.4.3. Storage

This capability refers to an IoE system’s storage function, based on the paradigm where its storage function resides: cloud, fog, or edge [73]. A storage platform (public, virtual, or private) offers the flexibility and scalability that an IoE application needs, from development to deployment [29]. Storage refers to storing data internally, and it varies intensively from one object to another [55]. Storage interactions between IoE enablers may be distinguished significantly depending on the object’s storage capabilities. Some objects may have restricted capabilities and store minimum information [132]. Most mobile devices at the edge of the network are resource-constrained in terms of storage, computation capability and battery life [56]. Although almost all of the objects have the capacity to store embedded codes to function internally, they differ in storing aggregated and processed data [104]. An object’s storage should also be based on the sensitivity of the information stored [55].

Analytics processing requires real-time data stream processing for supporting the rate of data arrival, data management, and data storage [105] at diverse distribution levels.

Depending on the storage and compute capabilities, the storage capability of an IoE node or application is [114]:

1. Device-level: devices are participants in the storage and compute process;
2. Network-level: the storage process uses remote connections to fog computing nodes;
3. Cluster level: storage function is provided between a set of interconnected servers [114].

## 5. Discussion and Comparison with Previous Work

In this section, we discuss theoretical and practical implications and limitations. We present a brief comparison of the scope of the proposed IoE taxonomy and 76 IoE and IoT taxonomy previous works selected in the literature review (presented in Section 3). We examined their diverse approaches in order to enhance understanding of the contextual aspects of IoE/IoT addressed and their relationships in identifying knowledge in IoE/IoT applications.

Table 2 shows the adherence of the analyzed studies to our proposed IoE taxonomy across the proposed categories and dimensions. In relation to dimensions of the IoE taxonomy, *capabilities* is the category most frequently addressed and studied, followed by *observation* and *type* of sensor, respectively. The summaries show that most taxonomies support at least two dimensions, but *knowledge* support is limited.

The proposed IoE taxonomy (in bold) covered all (100%) of the 18 dimensions. It should be noted that, on average, the remaining 76 studies covered 25,5% of the dimensions. The framework proposed by Boyes and Hallaq [113] obtained the second-highest coverage (72,2%), with 13 dimensions; however, it did not include aspects related to the type of knowledge in IoE applications. On average, the *knowledge* category obtained 24,7% coverage, while the *type* of sensor, *observation*, and *capabilities* categories appeared in 20.5, 20, and 44.3% of the studies, respectively.

The results indicated a lack of interest (only 15.8%) in identifying knowledge sources in terms of explicitness (tacit, explicit, or implicit). Moreover, only 13.1% of the studies addressed how the outcome of the IoE application was achieved and benefited by complementation (accompaniment) or substitution (replacement) of knowledge in IoE processes (between things, data, and humans). Thus, further research should consider this gap and attempt to examine the impact of knowledge identification on the design of IoE applications and how knowledge should be synthesized and combined to drive knowledge creation and intelligent services that create value. In conclusion, the findings of this present study provided an insight into the current trend of IoE research.

## 6. Results

This section presents the application of the proposed taxonomy in diverse IoE domains. We intended to validate the IoE taxonomy’s practical applicability for classifying knowledge in the IoE applications in relation to the following proposed categories: knowledge, type, observation, and capabilities. We conducted conceptual and pragmatical validations aimed to show that the proposed taxonomy involves the qualitative attributes of robustness and comprehensiveness. It contains enough dimensions and characteristics to clearly differentiate the objects of interest into distinct domains and to classify all known objects within the field under consideration [88].

### 6.1. Validation of Proposed IoE Taxonomy in Distinct Domains

We illustrate the validation of the proposed taxonomy over 50 distinct IoE applications in the following three domains: crowdsourcing applications [134], IoT/IoE applications with analytics [114], and cyber-physical systems [135]. We selected crowdsourcing applications due to the integration of crowd knowledge and participatory sensing, IoT/IoE applications with analytics to validate the applications with an implicit knowledge and big data sensing and cyber-physical systems due to the pervasive environment of thing-to-thing collaborations.

Table 3 presents a sample of analyses of the 50 applications. Our analyses will provide the roadmap for future research on IoE sensors and applications. To assist with future studies, the full results and details are available in a dataset within a technical report [136] (https://www.cos.ufrj.br/uploadfile/publicacao/2963.pdf).

Using different perspectives and approaches, most IoE applications demand knowledge, such as knowledge provided by sensors, knowledge about system’s domains, knowledge about users and activities, as well as knowledge for automated configuration of sensors and data annotation, reasoning, and event detection to the IoE system [93].

Regarding the crowdsourcing application domain and using the proposed taxonomy, we analyzed 11 applications observed by Melo et al. [134] in a Crowd Application Database (http://cadb.demoro.net). According to the authors of this study, there is a need to create mechanisms to warn users about using their data. It is essential to evaluate the boundaries between people and things and their collaboration processes to create collective intelligence. This should benefit from our IoE taxonomy in terms of knowledge identification and awareness. The 11 knowledge-intensive applications selected from the crowd application database were: Noisetube [137], CenceMe [138], MicroBlog [139], Ubifit Garden [140], GarbageWatch [141], Galaxy Zoo [142], eBird [143], SenSay [144], Jog Falls [145], MobAsthma [146], and Transafe [147]. These applications are intrinsically composed of knowledge-intensive tasks for the expected purpose and value creation. The transformations or automation provided by these applications consist of conversions of tacit-explicit-implicit knowledge when people, things, and data are connected in the IoE environment to provide relevant services and collective intelligence. Table 3 classifies these 11 applications using the proposed IoE taxonomy. The full results and details are available in [136].

Regarding IoE applications that benefit from data analytics, we selected 30 applications in Siow et al. [114], which analyzed (for the 2011–2017 period) the top five application domains: health, living, environment, industry, and transport. We categorized the selected applications (from distinct domains and with diverse analytical capabilities) in order to validate the IoE taxonomy, as presented in [136].

As a sample for application of the proposed IoE taxonomy, Table 3 shows the categorization of 5 applications that were selected from Siow et al. [114] (smart clothing monitoring [148], travel routing [149], chemical process monitoring [150], smart farming [151], and on-shelf availability [152]). We prioritized applications with descriptive capabilities, which are the primary source of knowledge creation. The five applications consist of knowledge sharing between sensors’ explicit knowledge, tacit human knowledge, and implicit knowledge in systems.

Smart clothing monitoring [148] is a monitoring system improved with a machine-learning algorithm for diagnostic and predictive analytics of patients’ health conditions. Travel routing [149] suggests the best travel routes, using analytical techniques for traffic flow prediction in order to predict future traffic flow. Chemical process monitoring [150] uses predictions to provide quality monitoring and enhanced control systems in plants to automatically react and prescribe process improvements “to prevent off-grade products”. Smart farming [151] applications discover relevant events on semantically enriched data streams from sensors related to two smart farming scenarios. Moreover, on-shelf availability [152] is a system that improves shoppers’ experiences, forecasts demand and provides insights on buyers’ behavior.

Siow et al. [114] emphasized similarities between knowledge hierarchy, which transforms data to wisdom with analytical capabilities of IoE applications. Their study formed a comprehensive hierarchical classification of analytic capabilities (descriptive, diagnostic, discovery, predictive, and prescriptive), where each level of the hierarchy lies in the previous tier and relates to a corresponding level of data-to-knowledge-wisdom hierarchy approach. However, the study neglected the characteristics of knowledge processes and combination of tacit (from humans), explicit (sensed data), and implicit (analytics) knowledge, which we consider to be the great impact for knowledge created from analytics capabilities and value creation from intelligent services. We addressed this understanding in the proposed knowledge-based IoE taxonomy, which characterizes knowledge sources in terms of the smart services they provide, their characteristics, and how they interact to provide foresight: real-time data (explicit knowledge) combines with models from learning systems (implicit knowledge) to recommend action (integrating tacit knowledge from human sensors).

Additionally, we used the proposed IoE taxonomy to understand nine domains of cyber-physical systems (CPS) [135]; the full results and details are available in a dataset within a technical report [136]. In Table 3, we validated the IoE taxonomy in terms of the main characteristics of CPS applications [135]. CPS applications incorporate physical processes, highly networked computers, machines, and robots to interact with the physical world in an extremely integrated and technical environment. They have a vital impact on older people’s daily lives, as well as on healthcare, agriculture, manufacturing, energy and critical infrastructures, logistics, transport, security, and safety. In applications that support older people’s daily lives, patient care services will improve their quality. They will benefit from the combination of real-time data collected from wearable sensors with tacit knowledge from medical professionals and AI from specialized task-oriented robots. In healthcare application domains, robots and humans will work together in a smart medical environment, and diagnostic processes based on evidence-supported results and treatments will be automated and optimized. In agriculture, information mining and decision-making patterns will cause job losses as technologies replace human workers. Even though industries will develop autonomous agricultural machines, this will create a niche for high-skilled jobs.

The proposed knowledge-based taxonomy in the classification of CPS enablers, in many ways, will support a foundation to build integration of tacit knowledge from human sensors in knowledge-intense applications applied to cyber-physical environments such as in CPS.

The recognized value of tacit knowledge from humans will emerge in highly trained skilled engineers who will manage robots. In manufacturing, there will be an increasing incentive for those who acquire digital skills to deal with a vast volume of real-time data collected through sensors.

### 6.2. Example of Classification of One Application with the Proposed Taxonomy

Table 4 describes the classification of a specific application selected from Siow et al. [114] in accordance with the previously defined taxonomy. We selected the on-shelf availability application [152] application due to the diversity of its sensor types and knowledge sources.

The on-shelf availability application [152] is an industry domain application [114] and relates to a system that benefits customers’ experience by enhancing the on-shelf availability of products. The system also seeks to forecast demand and provide insights into buyers’ behavior, with predictive analytics employed in business processes. It is composed of an effective algorithm that benefits from sensor data and provides a wide scope for discovering patterns and trends. Real-time data from sensors provides the relevant information to actuators (staff of the store) to immediately solve problems like products being out of stock on the shelves.

Other sensors like video cameras process video streams locally to provide the analysis of product availability on the shelves. The information is confirmed by other sensors (light sensors, infra-red, and RFID sensors), and the data and metadata are sent to the cloud to be processed. Other relevant information, such as weather data, local events and commemorative dates, and promotion details, are analyzed and combined with the current on-shelf availability of products to provide demand forecasting and model buyer behavior [114]. In the cloud servers, real-time data are processed and combined with models from learning systems, data obtained from enterprise Point of Sale systems, and inventory systems to recommend action plans to maintain the on-shelf availability of products. The store staff is informed, and action is taken to restock products and rectify business processes for quality improvement. While shopping, customers can take their pets to the pet store for bathing and grooming, and they can use their mobile devices to monitor their pets via a sensor on the animal’s collar.

The classification of the on-shelf availability application using the proposed IoE taxonomy highlighted the interdependencies between knowledge characteristics, sensors, and observations, considering the capabilities of available resources and expected outcomes for a new buying experience, such as insights for customers and business process improvement for suppliers.

With these elements, we defined a common vocabulary that can uncover existing and forthcoming application characteristics. The taxonomy has the quality attribute of being concise and having a limited number of dimensions, restricted to what is relevant and understandable. Moreover, it is explanatory enough to provide useful explanations and valuable descriptions of the nature of the exemplified application selected.

## 7. Conclusions

This work contributes to the development of a knowledge-based taxonomy related to IoE applications, which will guide both interested researchers in this field, as well as application developers, in the design of knowledge-intensive IoE services.

The proposed taxonomy is extendable: it allows for the inclusion of additional dimensions and new characteristics within the IoE paradigm and other emerging paradigms under the IoE umbrella or concerned with intelligent network connections.

The novel knowledge-based IoE taxonomy was revealed considering diverse approaches and main findings in 76 relevant works selected from the literature review of IoT and IoE taxonomies, which provided guidelines and a comprehensive overview of the topic. In Table 2, we presented the analyzed studies’ adherence to our proposed IoE taxonomy, which is proved to be the most comprehensive taxonomy taking into consideration the other taxonomies compared.

The conceptual and pragmatical validations aimed to show that the proposed taxonomy involves the qualitative attributes of robustness and comprehensiveness and contain enough dimensions and characteristics to differentiate the objects of interest from distinct application domains clearly: cyber-physical systems (CPS) [135], crowdsourcing applications [137,138,139,140,141,142,143,144,145,146,147], applications with analytics: [148,149,150,151,152], as presented in Table 3.

To exemplify the orchestration of intelligence in network connections concerning knowledge processes, type of IoE enablers, observation characteristics, and technological capabilities in IoE applications, Table 4 presents the IoE enablers’ classification and specific industry domain application [114], according to IoE proposed taxonomy characteristics. With this study, we aimed to better understand the potential in reshaping interactions among people and things in the IoE context, considering a knowledge management perspective. In future work, we will extend the research and apply a quantitative assessment for ranking knowledge of IoE enablers, based on the hierarchical structure of the proposed IoE taxonomy.

Nevertheless, in order to entirely understand the transformative potential of collaboration between people and things in IoE applications, there is a research gap that must be overcome regarding insights into the characteristics of knowledge creation, actions, and transformations provided by using IoE applications and the value created from people and things in this context. Thus, we believe there is still significant room for future research and work on this topic.

## Figures and Tables

**Table 1:** Summary of literature review stages.

Literature Review Stage	Number of Papers
Search of ISI Web of Science	235
Search of Scopus	323
Search of IEEE	118
Search of ACM Digital Library	22
Science@Direct	62
Total	760
Duplicates	366
Total after discarding duplicates	394
Approval for analytical reading	76
Rejected	318

**Table 2:** Comparison of the scope of the proposed IoE taxonomy with previous works.

Category		Knowledge					Type					Observation					Capabilities			Score
Dimensions		Explicitness	Structure	Trust	Outcome	Action	Presentation	Nature	Use	Role	Engagement	Location	Reach	Mobility	Time	Mode	Communication	Processing	Storage	Total Acquired
Ref.	Year																			
This study	2020	✓	✓	✓	✓	✓	✓	✓	✓	✓	✓	✓	✓	✓	✓	✓	✓	✓	✓	100%
[24]	2019					✓		✓	✓	✓		✓			✓		✓			38.8%
[26]	2019									✓		✓		✓			✓	✓		27.7%
[27]	2019		✓	✓													✓			16.6%
[30]	2019						✓	✓						✓			✓	✓	✓	33.3%
[57]	2019				✓	✓					✓						✓	✓		27.7%
[61]	2019			✓													✓			11.1%
[39]	2019			✓		✓	✓			✓				✓	✓		✓	✓	✓	50%
[64]	2019		✓	✓					✓											16.6%
[65]	2019			✓	✓		✓		✓		✓		✓		✓	✓	✓			50%
[69]	2019		✓	✓		✓									✓	✓	✓			33.3%
[84]	2019				✓		✓													11.1%
[95]	2019	✓	✓				✓													16.6%
[105]	2019		✓			✓												✓	✓	22.2%
[107]	2019		✓												✓	✓				16.6%
[109]	2019			✓		✓									✓					16.6%
[112]	2019			✓													✓	✓	✓	22.2%
[126]	2019													✓			✓	✓		16.6%
[62]	2018						✓		✓											11.1%
[37]	2018			✓				✓			✓				✓					22.2%
[55]	2018			✓	✓	✓												✓	✓	27.7%
[56]	2018			✓														✓	✓	16.6%
[59]	2018						✓			✓							✓	✓	✓	27.7%
[60]	2018			✓		✓						✓	✓	✓	✓		✓	✓	✓	50%
[49]	2018			✓											✓		✓			16.6%
[50]	2018		✓						✓								✓			16.6%
[51]	2018				✓	✓	✓			✓				✓			✓			33.3%
[76]	2018	✓	✓																	11.1%
[80]	2018											✓	✓	✓			✓			22.2%
[81]	2018											✓	✓	✓						16.6%
[83]	2018						✓	✓									✓			16.6%
[99]	2018	✓	✓											✓			✓	✓		27.7%
[102]	2018	✓	✓																	11.1%
[104]	2018		✓	✓	✓	✓			✓								✓	✓	✓	44.4%
[106]	2018		✓	✓		✓		✓	✓	✓	✓						✓	✓	✓	55.5%
[113]	2018			✓	✓	✓		✓	✓	✓		✓	✓	✓	✓		✓	✓	✓	72.2%
[114]	2018		✓	✓						✓							✓	✓	✓	33.3%
[116]	2018			✓								✓	✓				✓			22.2%
[77]	2018				✓					✓				✓			✓			22.2%
[120]	2018							✓			✓	✓			✓	✓				27.7%
[124]	2018											✓	✓				✓	✓	✓	27.7%
[127]	2018																✓	✓		11.1%
[25]	2017				✓	✓											✓	✓		22.2%
[41]	2017	✓															✓	✓	✓	22.2%
[42]	2017	✓	✓			✓			✓		✓									27.7%
[43]	2017								✓					✓			✓	✓		22.2%
[47]	2017			✓								✓					✓			16.6%
[63]	2017	✓			✓		✓	✓	✓		✓									33.3%
[48]	2017								✓	✓		✓		✓			✓	✓		33.3%
[40]	2017		✓	✓		✓												✓	✓	27.7%
[75]	2017								✓			✓		✓				✓	✓	27.7%
[79]	2017						✓		✓	✓		✓	✓		✓					33.3%
[94]	2017	✓	✓									✓	✓	✓	✓		✓	✓	✓	50%
[101]	2017	✓	✓	✓		✓														22.2%
[133]	2017		✓																	5.5%
[29]	2016								✓									✓	✓	16.6%
[44]	2016								✓	✓				✓			✓			22.2%
[66]	2016	✓					✓		✓			✓								22.2%
[72]	2016								✓	✓		✓				✓				22.2%
[73]	2016		✓															✓	✓	16.6%
[111]	2016			✓													✓	✓	✓	22.2%
[115]	2016			✓													✓	✓	✓	22.2%
[118]	2016					✓											✓			11.1%
[28]	2015		✓				✓		✓	✓			✓	✓		✓	✓			44.4%
[53]	2015			✓			✓										✓			16.6%
[67]	2015						✓		✓		✓									16.6%
[71]	2015		✓						✓				✓							16.6%
[74]	2015		✓				✓						✓			✓	✓	✓	✓	38.8%
[82]	2015	✓										✓		✓	✓	✓				27.7%
[45]	2014			✓						✓		✓					✓			22.2%
[36]	2014	✓	✓			✓	✓			✓		✓	✓	✓	✓		✓			55.5%
[108]	2014			✓													✓			11.1%
[110]	2014			✓				✓	✓						✓		✓			27.7%
[35]	2013						✓	✓		✓										16.6%
[68]	2013		✓	✓		✓									✓		✓	✓		33.3%
[46]	2011			✓			✓	✓		✓										22.2%
[38]	2011																✓	✓	✓	16.6%

**Table 3:** Validation of proposed IoE taxonomy in distinct domains.

Category/Dimension		Applications Classified According to IoE Proposed Taxonomy Characteristics: Cyber-Physical Systems (CPS) [136], Crowdsourcing Applications [137,138,139,140,141,142,143,144,145,146,147], Applications with Analytics: [148,149,150,151,152]
Knowledge	Explicitness	**Tacit** [114,137,138,139,140,144,145,146,147,151,152] **Explicit** [136,138,139,140,142,143,144,146,147,150,151,152] **Implicit** [136,141,145,146,149,150,151,152]
	Structure	**Structured** [135,137,138,139,140,141,142,143,144,145,146,147,148,149,150,151,152] **Semi-structured** [135,146,147,152] **Unstructured** [135,145]
	Trust	**Trustful** [135,148,149,150,151,152] **Untrustful** [137,138,139,140,141,142,143,144,145,147]
	Outcome	**Complements** [135,137,138,139,140,141,142,143,144,145,146,147,148,149,150] **Substitutes** [135,151,152]
	Action	**Automation** [135,137,150,151,152] **Transformation** [135,138,139,140,141,142,143,144,146,147,148,149]
Type	Presentation	**Cyber** [135] **Physical** [135,137,138,139,142,143,144,145,146,147,148,149,150,151,152] **Cyber-physical** [135,140,142,144,145,146,147,148,149,150,151,152]
	Nature	**Electronic-based** [135,137,148,149,150,151,152] **Software-based** [135,147,150] **Human-based** [135,137,138,139,140,141,142,143,144,145,146,147,151,152]
	Use	**Wearables** [135,137,138,139,140,141,142,152] **Surroundable** [135,148,149,150,151] **Embeddable** [140,142,150]
	Role	**Sensor** [137,138,139,140,141,142,143,144,145,146,147,149,152] **Actuator** [152] **Sensor and actuator** [135,148,150,151]
	Engagement	**Opportunistic** [135,140,144,146,149,151] **Participatory** [141,142,143,145,147,148,150,152]
Observation	Location	**Coarse-grained** [137,138,139,141,142,143,144,147,148,149,150,151] **Fine-grained** [135,140,145,146,152]
	Reach	**Full** [137,138,139,141,142,143,144,145,147,148] **Partial** [135,140,146,150,151]
	Mobility	**Fixed** [152] **Mobile** [137,138,139,140,141,142,143,144,145,146,147,148,149,150,151]
	Time	**Pull** [140,145,147,148,149,150,152] **Push** [135,137,138,139,140,141,142,143,144,146,151,152]
	Mode	**Sense** [135,137,138,139,140,141,142,143,144,145,146,147,148,149,150,151,152] **Derive** [135,140,145,146,149,151,152] **Manually provided** [142,143,148]
Capabilities	Communication	**Semantic** [135,137,138,139,140,141,142,143,144,145,146,147] **Pragmatic** [135,148,149,150,151] **Conceptual** [152]
	Processing	**Cloud** [135,137,138,139,140,141,142,143,144,145,146,147,148,149,150,151,152] **Fog mobile edge**: [139,140,144,145,147]
	Storage	**Device level** [150] **Network level** [149,152] **Cluster level** [135,137,138,139,140,141,142,143,144,145,146,147,148,149,150,151,152]

**Table 4:** Classification of IoE enablers of a specific industry domain application [114], according to IoE proposed taxonomy characteristics.

Category/Dimension		Characteristics of an industry domain application (on-shelf availability application [152])
**Knowledge**	**Explicitness**	**Tacit**: shoppers’ experience, staff experience | **Explicit**: enterprise point of sale (POS) systems and inventory systems | **Implicit**: algorithm and models from learning systems
	**Structure**	**Structured**: enterprise data| **Semi-structured**: weather data, local events, and promotion details | **Unstructured**: real-time sensor data
	**Trust**	**Trustful**: data from enterprise systems | **Untrustful**: real-time data from shoppers’ sensors
	**Outcome**	**Complements**: Recommended action plans | **Substitutes**: predictive analytics to provide insights
	**Action**	**Automation**: stock business processes | **Transformation**: insights into buyers’ behavior
**Type**	**Presentation**	**Cyber**: predictive analytics algorithm | **Physical**: cameras, shoppers, staff of the store, light, infra-red, and RFID sensors | **Cyber-Physical**: point of sale (POS) systems
	**Nature**	**Electronic-based**: video cameras, light, infra-red, and RFID sensors | **Software-based**: point of sale (POS) systems | **Human-based**: shoppers, the staff of the store | **Non-human-based**: shoppers’ pets
	**Use**	**Wearables**: shoppers’ mobile devices | **Surroundables**: video cameras, infra-red sensors | **Embeddable**: light, RFID sensors
	**Role**	**Sensor**: video cameras, light, infra-red, and RFID sensors, shoppers, the staff of the store | **Actuator:** staff of the store who restock products or actuators to rectify problems | **sensor, and actuator**: staff of the store who senses and executes recommended actions
	**Engagement**	**Opportunistic**: shoppers | **Participatory**: shoppers/staff of the store
**Observation**	**Location**	**Coarse-grained**: supply chain context | **Fine-grained**: store environment
	**Reach**	**Full**: supply chain context **Partial**: physical store environment
	**Mobility**	**Fixed**: inside the store supply chain context | **Mobile**: shoppers’ mobile devices
	**Time**	**Pull**: meta-data produced and sent to the cloud | **Push**: forecast demands provided by systems
	**Mode**	**Sense**: store sensor devices | **Derive**: information derived from sensors |**Manually provided**: data provides from shoppers’ demand
**Capabilities**	**Communication**	**Conceptual communication**: supports the execution of recommended actions and provides a novel shopping experience
	**Processing**	**Cloud**: metadata produced | **Fog/Edge**: Edge: video streams processed locally | **Mobile cloud**: mobile devices from shoppers
	**Storage**	**Device-level**: processing video streams locally | **Network level** | **Cluster level**: metadata produced is sent to the cloud

## Data Availability

Not applicable.
